# Tumour assessment and staging: United Kingdom National Multidisciplinary Guidelines

**DOI:** 10.1017/S002221511600044X

**Published:** 2016-05

**Authors:** N Roland, G Porter, B Fish, Z Makura

**Affiliations:** 1 Department of ENT – Head & Neck Surgery, University Hospital Aintree, Liverpool, UK; 2 Department of ENT – Head & Neck Surgery, University Hospitals Bristol, East Grinstead, UK; 3 Department of Otolaryngology – Head & Neck Surgery, Cambridge University Teaching Hospitals Trust, East Grinstead, UK; 4 Head & Neck Unit, Queen Victoria Hospital NHS Foundation Trust, East Grinstead, UK

## Abstract

In general, the first decision to be made in a patient with a confirmed head and neck cancer is whether or not to treat the patient before deciding what form of management strategy is appropriate. There is no more important an aspect of head and neck cancer care than the initial evaluation of the patient and the patient's tumour. The practice requires specific expertise and judgement. The current tumour–node–metastasis system relies on morphology of the tumour (anatomical site and extent of disease) but the final decision on treatment hinges on a full assessment of the patient including physiological age and general condition. The aim of this paper is primarily to describe why and how we appraise a patient and their tumour. It addresses the general principles applicable to the topic of evaluation, classification and staging. In addition, the limitations and pitfalls of this process are described.

**Recommendations:**

• All patients with head and neck cancer (HNC) should undergo tumour classification and staging prior to treatment. (R)

• Pre-therapeutic clinical staging of HNCs should be based on at least a C2 factor (evidence obtained by special diagnostic means, e.g. radiographic imaging (e.g. computed tomography, magnetic resonance imaging or ultrasound scan), endoscopy, biopsy and cytology). (R)

• Imaging to evaluate the primary site should be performed *prior* to biopsy to avoid the effect of upstaging from the oedema caused by biopsy trauma. (G)

• Panendoscopy is only recommended for symptomatic patients or patients with primary tumours known to have a significant risk of a second (synchronous) primary tumour. (G)

## Introduction

There are many aspects affecting the outcome of patients with malignant head and neck tumours. These may relate to the tumour (e.g. the anatomical site and extent of the disease), the host (age, general condition and any concurrent disease) and management (treatment options, expertise available and patient preference).

Staging of head and neck cancer (HNC) is a system designed to express the relative severity, or extent, of the disease. The objectives are illustrated in Table 1.

**Table I:** Objectives of staging

1. To aid the clinician in the planning of treatment 2. To give some indication of prognosis 3. To assist in evaluation of the results of treatment 4. To facilitate the exchange of information between treatment centres 5. To contribute to the continuing investigation of human cancer

The nature of staging has meant that the data to support the concept have been largely drawn from retrospective and observational studies. Much of the systems development has been through the opinion of expert panels using these data.

Both the International Union against Cancer (UICC) and the American Joint Committee on Cancer (AJCC) published rules on classification and staging which correspond in their 7th editions (2009) and have approval of all national tumour–node–metastasis (TNM) committees.^1^^,^^2^

## Sites in the head and neck region

The TNM classification applies only to carcinomas and melanomas in the following sites: lip and oral cavity, pharynx (oropharynx, nasopharynx and hypopharynx), larynx, maxillary sinus, nasal cavity and ethmoid sinus, mucosal malignant melanoma, major salivary glands and thyroid gland. Each site is described having rules for classification, anatomical sites and subsites where appropriate, the clinical TNM (cTNM) classification, the pathological TNM (pTNM) classification, G histopathological grading, stage grouping and a summary. The main aspects are described here, but specific details can be found in the most recent UICC and AJCC TNM booklets.^1^^,^^2^

## General rules

The TNM system for describing the anatomical extent of the disease is based on three components (Tables II–IV):

**Table II:** An overview of the tnm staging terminology

T – Primary tumour			
TX		Primary tumour cannot be assessed	
T0		No evidence of primary tumour		
Tis		Carcinoma in situ	
T1, T2, T3, T4		Increasing size and/or local extent of the primary tumour	
N – Regional lymph nodes			
NX		Regional lymph nodes cannot be assessed	
N0		No evidence of regional lymph node metastases	
N1, N2, N3		Increasing involvement of regional lymph nodes	
M – Distant metastasis			
M0		No distant metastasis	
M1		Distant metastasis	
The previously included MX category is now considered to be inappropriate.			
The category M1 may be further specified according to the following notation:			
Pulmonary	PUL	Bone marrow	MAR
Osseous	OSS	Pleura	PLE
Hepatic	HEP	Peritoneum	PER
Brain	BRA	Adrenals	ADR
Lymph nodes	LYM	Skin	SKI
Other	OTH

**Table III:** Histopathological grading system for squamous cell carcinoma

GX	Grade of differentiation cannot be assessed
G1	Well differentiated
G2	Moderately differentiated
G3	Poorly differentiated
G4	Undifferentiated

G = Histopathological grading

**Table IV:** Optional descriptors used for histopathological reporting in squamous cell carcinoma

Optional descriptors
Pn – Perineural invasion	
PnX	Perineural invasion cannot be assessed
Pn0	No perineural invasion
Pn1	Perineural invasion
L – Lymphatic invasion	
LX	Lymphatic invasion cannot be assessed
L0	No lymphatic invasion
L1	Lymphatic invasion
V – Venous invasion	
VX	Venous invasion cannot be assessed
V0	No venous invasion
V1	Microscopic venous invasion
V2	Macroscopic venous invasion

T – Extent of the primary tumour

N – Absence or presence and extent of regional lymph node metastases

M – Absence or presence of distant metastases

All cases should be confirmed microscopically. Two classifications should be documented for each site, namely: cTNM (clinical (pre-treatment) classification) and pTNM (post-surgical histopathological classification). The clinical stage is essential to select and evaluate therapy, while the pathological stage provides the most precise data to estimate prognosis and calculate end results. It should be remembered that if there is doubt concerning the correct T, N or M category to which a particular case should be allotted, then the *lower* (*i.e. less advanced*) category should be chosen. After assigning the cTNM and pTNM categories, the patient should then be classified in a Stage Group. Once established, this must remain unchanged in the medical records.^1^^,^^2^

See site-specific chapters for each detailed tumour classification.

## Histopathological grading

The histological grading of squamous cell carcinoma represents estimation by the pathologist of the expected biologic behaviour of the neoplasm. Although it is subject to inter- and intra-observer errors, it has been suggested such information in conjunction with other characteristics of the primary tumour is useful in the rational approach to therapy.^3^ The grade can be applied to all head and neck sites except thyroid.

## Additional descriptors

Designation is now applicable when sentinel lymph node biopsy is attempted using the suffix (sn) after N stage. Optional descriptors for perineural invasion (Pn), lymphatic invasion (L) and venous invasion (V) may be used.

The absence or presence of residual tumour after treatment may be described by the symbol R. A recurrent tumour, when classified after a disease-free interval is identified by the prefix ‘r’. The prefix ‘a’ indicates that classification is first determined at autopsy. The suffix ‘m’ is used to indicate the presence of multiple primary tumours at a single site. In cases where multimodality treatment is used, the cTNM or pTNM is identified by a ‘y’ prefix which categorises the extent of tumour actually present at the time of that examination.

The C-factor, or *certainty factor*, reflects the validity of classification according to the diagnostic methods employed (C1–C5). C1 would be evidence from standard diagnostic means whereas C5 is evidence from autopsy. Generally speaking, pre-therapeutic clinical staging of HNCs is equivalent to C1, C2 and C3, whilst pathological classification is equivalent to C4.^1^^,^^2^

## Related classifications

The World Health Organization (WHO) has developed a series aimed at classification of tumours. The WHO International Classification of Diseases for Oncology (ICD-O) is a coding system for neoplasms by topography and morphology and for indicating behaviour (e.g. malignant and benign).^4^ This coded nomenclature is identical in the morphology field for neoplasms to the Systemised Nomenclature of Medicine.^5^ It is recommended that the WHO classification of tumours is used for classification and definition of tumour types and that the ICD-O code is used for storage and retrieval of data.

## Stage grouping

After TNM, classification of tumours should be assigned a stage grouping between 0 or I and IV (Tables V). The grouping adopted is designed to ensure, as far as possible, that each group is more or less homogeneous in respect of survival and that the survival rates for each cancer stage are distinctive. Carcinoma in situ is categorised as stage 0; cases with distant metastasis as stage IV. The exceptions to this grouping are for carcinoma of the nasopharynx, carcinoma of the thyroid (Tables VI and VII) and mucosal melanoma.^1^^,^^2^

**Table V:** Stage grouping for head and neck cancers excluding nasopharynx, thyroid and mucosal melanoma

Stage 0	Tis	N0	M0
Stage I	T1	N0	M0
Stage II	T2	N0	M0
Stage III	T1, T2, T3	N1	M0
	T3	N0	M0
Stage IVA	T1, T2, T3	N2	M0
	T4a	N0, N1, N2	M0
Stage IVB	Any T	N3	M0
	T4b	Any N	M0
Stage IVC	Any T	Any N	M1

**Table VI:** Stage grouping for carcinoma of the nasopharynx

Stage 0	Tis	N0	M0
Stage I	T1	N0	M0
Stage II	T1	N1	M0
	T2	N0,N1	M0
Stage III	T1,T2	N2	M0
	T3	N0, N1, N2	M0
Stage IVA	T4	N0, N1, N2	M0
Stage IVB	Any T	N3	M0
Stage IVC	Any T	Any N	M1

**Table VII:** Stage grouping for thyroid carcinoma

Papillary or follicular *under 45 years*			
Stage I	Any T	Any N	M0
Stage II	Any T	Any N	M1
Papillary or follicular *45 years and older*			
Stage I	T1a, T1b	N0	M0
Stage II	T2	N0	M0
Stage III	T3	N0	M0
	T1, T2, T3	N1a	M0
Stage IVA	T1, T2, T3	N1b	M0
Stage IVB	T4a	N0, N1	M0
Stage IVC	T4b	Any N	M0
	Any T	Any N	M1
Medullary			
Stage I	T1a, T1b	N0	M0
Stage II	T2, T3	N0	M0
Stage III	T1, T2, T3	N1a	M0
Stage IVA	T1, T2, T3	N1b	M0
Stage IVB	T4a	Any N	M0
Stage IVC	T4b	Any N	M0
	Any T	Any N	M1
Anaplastic (all cases are stage IV)			
Stage IVA	T4a	Any N	M0
Stage IVB	T4b	Any N	M0
Stage IVC	Any T	Any N	M1

Separate stage groupings are recommended for papillary and follicular, medullary and undifferentiated carcinomas

## Methods of assessment

The aim is to define in each patient all of the factors relevant to the natural history and outcome of the relevant disease, thereby enabling a patient with cancer to be grouped with other similar cases. The sex and age of the patient, the duration and severity of symptoms and signs and the presence and severity of concurrent disease should all be documented.

Computed tomography (CT) and magnetic resonance imaging are now established as the mainstay investigations in the pre-operative work-up of patients with HNC, to delineate the extent and size of the primary tumour, to determine the presence (particularly when risk of occult nodes is >20 per cent), number and position of cervical lymph nodes, to search for an occult primary and to locate a synchronous primary or distant metastases (particularly the chest). Appropriate screening for synchronous tumours and distant metastases is particularly important in advanced tumours. Several studies have suggested that a CT scan should be obtained in preference to a plain chest X-ray as this may miss significant lung pathology.^6^ There is a growing body of evidence that points to the value of 18F fluoro-deoxyglucose-positron emission tomography/CT in the management of HNC patients and predicting patient-related outcomes. It is invaluable in the detection of the unknown primary and useful in the confirmation of residual or recurrent disease, but is not routinely used in initial staging assessment.^7^

Endoscopy and biopsy should be performed by a senior surgeon and in *all cases* by the head and neck surgeon responsible for any future procedure. This should include for each tumour a description, diagrammatic representation and preferably also photographic documentation. Routine panendoscopy (oesophagoscopy and bronchoscopy) is contentious. Proponents point out that these procedures require very little time, and may be performed easily during planned, direct laryngoscopy. A large meta-analysis found a small advantage to panendoscopy in detection of second primary tumours during analysis of multiple prospective studies.^8^ Opponents point out that the appropriate use of symptom directed investigations in addition to routine chest radiography have a similar detection rate compared with screening endoscopy and avoid unnecessary risk and expense in asymptomatic patients.^9^ McGarey *et al*.^10^ concluded that while rigid oesophagoscopy is safe, the utility is low for cancer staging and for detection of non-malignant oesophageal disease. Review of the literature and analysis of a large national cancer dataset indicate that the incidence of synchronous oesophageal malignant neoplasms in patients with head and neck squamous cell carcinoma is low and has been decreasing during the past three decades.^10^ Thus, screening oesophagoscopy should be limited to patients with  head and neck squamous cell carcinoma who are at high risk for synchronous oesophageal malignant neoplasms.

There is a natural desire to confer a stage on the tumour at presentation in the clinic and certainly after endoscopy. This should be avoided. It is better to rely on descriptive text to avoid changing the stage as more information becomes available. The clinical (pre-treatment) classification (cTNM) based on examination, imaging, endoscopy and biopsy should be clearly documented in the case-file only when all of the above information is collated. The UICC book should be available in every theatre and clinic to assist in applying the *correct stage.*

## Regional lymph nodes

The status of the regional lymph nodes in HNC is of such prognostic importance that they must be assessed for each patient and tumour. Lymph nodes are described as ipsilateral, bilateral, contralateral or midline; they may be single or multiple and are measured by size, number and anatomical location (Table VIII). Midline nodes are considered ipsilateral nodes except in the thyroid. Direct extension of the primary tumour into lymph nodes is classified as lymph node metastasis.^1^^,^^2^

**Table VIII:** *N* STAGING FOR REGIONAL LYMPH NODES

NX	Regional lymph nodes cannot be assessed
N0	No regional lymph node metastasis
N1	Metastasis in a single ipsilateral lymph node. 3 cm or less in greatest dimension
N2	N2a Metastasis in a single ipsilateral lymph node, more than 3 cm but not more than 6 cm in greatest dimension
	N2b Metastasis in multiple ipsilateral lymph nodes, none more than 6 cm in greatest dimension
	N2c Metastasis in bilateral or contralateral lymph nodes, none more than 6 cm in greatest dimension
N3	Metastasis in a lymph node more than 6 cm in greatest dimension

Imaging for node detection and delineation is recommended in the following settings: the neck is being scanned as part of the evaluation of the primary tumour; there is a high chance of occult disease (e.g. supraglottic primary); to assess the extent of nodal disease; to define any deep nodal fixation; or if clinical assessment is difficult because of a short, fat or previously irradiated neck.

Lymph nodes are subdivided into specific anatomic sites and grouped into seven levels for ease of description. The pattern of lymphatic drainage varies for different anatomic sites. However, the location of the lymph node metastases has prognostic significance. Survival is significantly worse when metastases involve lymph nodes beyond the first echelon of lymphatic drainage.^11^ It is particularly poor for lymph nodes in the lower regions of the neck, i.e. levels IV and V (supraclavicular area).

International Union Against Cancer and AJCC recommend that each N-staging category be recorded to show, in addition to the established parameters, whether the nodes involved are located in the upper (U) or lower (L) regions of the neck, depending on their location above or below the lower border of the thyroid cartilage.^1^^,^^2^

The definitions of the N categories for all head and neck sites are the same (Table VIII) except thyroid (Table IX) and nasopharynx (Table X). The natural history and response to treatment of cervical nodal metastases from nasopharynx are different, in terms of their impact on prognosis, so they justify a different N classification. Regional lymph node metastases from well-differentiated thyroid cancer do not significantly affect the ultimate prognosis and therefore also justify a unique system.

**Table IX:** *N* Staging for thyroid carcinoma

NX	Regional lymph nodes cannot be assessed
N0	No regional lymph node metastasis
N1	Regional lymph node metastasis
N1a	Metastasis in level VI (pre-tracheal, pre-laryngeal, paralaryngeal) nodes
N1b	Metastasis in other unilateral, bilateral or contralateral cervical or retropharyngeal or superior mediastinal lymph node(s)

**Table X:** *N* Staging for nasopharynx

NX	Regional lymph nodes cannot be assessed
N0	No regional lymph node metastasis
N1	Unilateral cervical, unilateral or bilateral retropharyngeal lymph node(s), 6 cm or less in greatest dimension, above supraclavicular fossa
N2	Bilateral cervical lymph node(s), 6 cm or less in greatest dimension, above supraclavicular fossa
N3	Metastasis in lymph node >6 cm and/or (in the supraclavicular fossa:
(N3a)	Greater than 6 cm in dimension
(N3b)	In the supraclavicular fossa

Note: Midline nodes are considered ipsilateral nodes, and the supraclavicular triangle is defined by the lines joining the following three points – the superior margin of the clavicle at its sternal and acromial ends, and the point where the line of the neck meets the shoulder.

## Pathological classification (pTNM)

The pT, pN and pM categories correspond to the T, N and M categories, respectively. The extent of the tumour in terms of the location and level of the lymph node should be documented. In addition, the number of nodes that contain tumour and the presence or absence of extracapsular spread of the tumour should be recorded. Histological examination of a selective neck dissection including central compartment specimen usually includes six or more lymph nodes; a radical or modified radical neck dissection specimen includes 10 or more lymph nodes.^1^^,^^2^

The current TNM system relies on morphology of the tumour (anatomical site and extent of disease) with little or no attention given to patient factors. However, the literature does suggest that symptom severity^12^ and comorbidity^13^ have a significant impact on outcomes. It is therefore recommended that these data be recorded. Definitions of TNM categories may be altered or expanded for clinical or research purposes as long as the basic definitions are recorded and not changed. Despite the obvious value of staging, both in the management of individual patients and for the grouping of patients in trials and reports of treatment, it does have its limitations. The most insidious of these is that attempts to increase the accuracy of staging leads to greater complexity, and hence paradoxically to more errors and an increased likelihood of non-compliance by the person responsible for staging. Advances in methods of collecting and recording data will hopefully reduce these errors. Changes in the TNM classification should and will only occur, based on the appropriate collection, presentation and analysis of data, in the forum of the UICC and AJCC.^3^^,^^4^ The principles, practice and limitations of the current staging system are well documented in many major texts.^14^^–^^16^ Changes between editions tend to be conservative and commentaries regarding HNC reflect this.^17^ It is seven years since the 7th edition of the UICC and AJCC staging manuals and the updated version is eagerly awaited. The early indications are that changes will be only subtle and few.

### Key points

• Staging of head and neck cancer is a system designed to express the relative severity, or extent, of the disease. It is meant to facilitate an estimation of prognosis and provide useful information for treatment decisions. Classification by anatomical extent of head and neck cancer as determined clinically and histopathologically is the TNM System
• Radiological investigations to evaluate the primary site should be performed *prior* to biopsy to avoid the effect of upstaging from the oedema caused by biopsy trauma
• The sex and age of the patient, the duration and severity of symptoms and signs, and the presence and severity of inter-current disease should all be documented
• Assessment by endoscopy and biopsy should be performed by a senior surgeon and in all cases by the Head & Neck surgeon responsible for any future procedure
• The clinical (pre-treatment) classification (cTNM) based on examination, imaging, endoscopy and biopsy should be clearly documented in the case-file only when all the information is collated
• Individual TNM classifications should be assembled into four groups – stage groups (stages I–IV), each with similar survival outcomes
• The UICC book should be available in every theatre, MDT meeting and clinic to assist in applying the *correct stage*.
